# From Bench to Bedside: The Evolving Landscape of Stem Cell Therapies for Stroke Rehabilitation

**DOI:** 10.1155/sci/3734659

**Published:** 2025-12-28

**Authors:** Daniel Waszczuk, Shrivats Manikandan, Varsha Manikandan, Erian Stone, Shannon Buehre, Steiv Shore, Manikandan Panchatcharam, Sumitra Miriyala

**Affiliations:** ^1^ Kirksville College of Osteopathic Medicine, A.T. Still University, Kirksville, 63501, Missouri, USA, atsu.edu; ^2^ Department of Anatomy, Kirksville College of Osteopathic Medicine, A.T. Still University, Kirksville, 63501, Missouri, USA, atsu.edu; ^3^ Department of Chemistry, Truman State University, Kirksville, 63501, Missouri, USA, truman.edu; ^4^ Department of Neurology, Saint Louis University School of Medicine, St Louis, 63104, Missouri, USA, slu.edu

**Keywords:** angiogenesis, CRISPR/Cas9 gene editing, immunomodulation, ischemic stroke, neurogenesis, stem cell therapy

## Abstract

Globally, stroke stands as a principal cause of death and disability, presenting formidable challenges in rehabilitation. Conventional therapeutic modalities often fail to restore functional capabilities fully, underscoring the need for innovative treatment strategies. Stem cell therapy emerges as a revolutionary approach, capitalizing on the regenerative capabilities of stem cells to improve neurological function poststroke. This review evaluates the roles of various stem cell types—mesenchymal stem cells (MSCs), neural stem cells (NSCs), and induced pluripotent stem cells (iPSCs)—in the realm of stroke recovery. It elucidates their distinct biological mechanisms, evaluates their therapeutic impact based on clinical trial data, and discusses their efficacy in fostering neural repair and recovery. MSCs are particularly noted for their role in immunomodulation and promotion of angiogenesis and neurogenesis, with clinical evidence supporting their safety and effectiveness in stroke recovery. NSCs are lauded for their ability to differentiate into diverse neural lineages. They integrate into neural circuits to enhance synaptic connectivity and neuroplasticity. iPSCs, known for their versatility, can be tailored to patient‐specific needs and are shown in preclinical settings to reduce infarct size and promote the survival of neuronal cells. However, the field grapples with challenges, including optimizing stem cell transplantation timing, precision in cell delivery, integration efficiency, and immune system compatibility. These issues call for harmonization of methodologies across ongoing studies to ensure the reliability and consistency of therapeutic outcomes. This review highlights the promising future and challenges of stem cell therapy for treatment of stroke.

## 1. Introduction

Stroke is the second leading cause of death and disability globally, responsible for 11.6% of all deaths in 2019 [1–3]. The International Classification of Diseases (ICD‐11) in 2018 defines stroke as a neurological disorder precipitated by sudden disruptions in cerebral blood flow [1]. The condition leads to extensive multifunctional deficits including severe motor disabilities, cognitive impairments, sensory losses, and communication barriers [2]. Additionally, the psychosocial impact of stroke is profound; patients often endure vocational disruptions, social isolation, and a sense of dehumanization, contributing to feelings of hopelessness and worthlessness. The ripple effects extend to caregivers, who face heightened stress and financial strains, reflecting the broad societal and economic burdens of this condition [2]. Despite healthcare systems striving to mitigate the functional impacts of stroke, the psychosocial and educational needs of patients and their families frequently remain unaddressed.

Ischemic stroke (IS), the most prevalent form of stroke, affected approximately 77.2 million individuals in 2019, accounting for 63.5 million disability‐adjusted life years (DALYs) and 3.3 million deaths [1, 3]. While age‐standardized mortality and incidence rates of stroke have declined from 1990 to 2019, the total numbers of stroke‐related deaths and DALYs have surged, likely due to increased life expectancy and population growth [3, 4]. This trend poses a significant concern, particularly for regions with lower socio‐demographic indices and under‐resourced healthcare infrastructures. These figures are projected to continue to rise markedly, accentuating the imperative for innovative therapeutic strategies that can transcend the limitations of current treatments and offer substantive improvements in patient outcomes.

Recovery from stroke is typically categorized into distinct temporal phases: the hyperacute phase (within 24 h), the acute phase (up to 7 days), the early subacute phase (up to 3 months), the late subacute phase (4–6 months), and the chronic phase (beyond 6 months) [4]. Neurological recovery is most pronounced within the initial week poststroke, with motor functions often stabilizing and reaching a plateau by the end of the third month. Beyond 6 months, recovery generally stabilizes, indicating entry into the chronic phase. Enhancements in cognitive functions have been documented during this period [3]. The effectiveness of stem cell‐based interventions can be significantly modulated by patient‐specific factors such as age‐related changes in neuroplasticity and the presence of comorbidities, which pose critical challenges for translating preclinical success into robust clinical outcomes.

The therapeutic management of stroke targets enhancing neuroprotection, optimizing reperfusion strategies, and stimulating neuroplasticity [1]. Traditional modalities for stroke treatment seldom reverse the functional or neurological deficits sustained, which underscores the necessity for novel therapeutic approaches. In this regard, stem cell therapy has emerged as a promising alternative, offering the potential to replace damaged neural tissue and reestablish functional neural circuits. The safety and efficacy of various stem cells, including mesenchymal stem cells (MSCs), neural stem cells (NSCs), and induced pluripotent stem cells (iPSCs), have been validated in numerous preclinical and clinical trials. Leveraging the regenerative capabilities of these cells is seen as pivotal in enhancing patient outcomes and alleviating the escalating burden of stroke on global healthcare systems. The ongoing success and rigorous exploration of stem cell‐based techniques are thus critical to advancing stroke therapy. This review distinguishes itself by integrating mechanistic insights into blood–brain barrier (BBB) permeability, paracrine effects, and genetic editing approaches, alongside a synthesis of updated clinical trial data and the latest recommendations from the Stem Cells as an Emerging Paradigm in Stroke (STEPS) initiative. Together, this comprehensive approach provides a more holistic perspective compared to previous reviews.

## 2. Stem Cell Types in Stroke Treatment

As research in regenerative medicine advances, stem cell‐based therapies have emerged as promising candidates for addressing the complex pathophysiology of IS. Among the various cell types investigated, three have gained particular scientific and clinical attention: MSCs, NSCs, and iPSCs. Each possesses distinct biological characteristics, mechanisms of action, and therapeutic potential. The following sections provide an overview of these stem cell types, outlining their origins, mechanisms in stroke recovery, current clinical progress, and the key limitations that shape their translational prospects.

### 2.1. MSCs

MSCs are multipotent stromal cells with remarkable plasticity and immunomodulatory properties, making them invaluable in regenerative medicine, particularly for neurological applications. Derived from pericytes in various tissues such as bone marrow, adipose tissue, umbilical cord blood, and dental pulp, MSCs are distinguished by their low immunogenicity and extensive differentiation potential [5–7]. This allows them to differentiate into diverse cell lineages, including osteogenic, adipogenic, neurogenic, cardiogenic, and hepatogenic pathways, underpinning their versatility in therapeutic applications [7, 8]. In stroke treatment, MSCs exert therapeutic effects primarily through their secretome, which comprises a wide array of cytokines, growth factors, and chemokines. These bioactive molecules include vascular endothelial growth factor (VEGF), angiopoietin‐1 (Ang‐1), basic fibroblast growth factor (bFGF), insulin‐like growth factor 1 (IGF‐1), transforming growth‐factor beta‐1 (TGF‐*β*1), nerve growth factor (NGF), brain‐derived neurotrophic factor (BDNF), placental growth factor (PGF), stromal‐derived factor‐1 (SDF‐1/CXCL12), and various interleukins (ILs) such as IL‐6, IL‐8, IL‐10, and IL‐13 [8–13]. These factors are critical for modulating the poststroke microenvironment, enhancing endogenous repair mechanisms, and facilitating recovery processes such as angiogenesis, neurogenesis, and immunomodulation [8, 9]. MSCs interact with the innate and adaptive immune systems, a process mediated by their secretion of prostaglandin E2 (PGE2), indoleamine 2,3‐dioxygenase (IDO), and soluble human leukocyte antigen G5 (sHLA‐G5). These interactions help modulate the activity of various immune cells, including dendritic cells, macrophages, natural killer cells, neutrophils, and T‐cell subsets [8, 9]. This modulation is crucial in stroke, where the inflammatory response is pivotal to the progression of neural injury. Recent meta‐analyses confirm MSCs’ robust immunomodulatory potential, but donor variability and cell heterogeneity remain key standardization challenges [14].

Numerous clinical trials have investigated MSCs for their potential in treating IS. According to the clinicaltrials.gov database, there are currently 32 clinical trials involving MSCs, with the majority being early‐phase trials (Phase I and II). This highlights the growing interest and perceived therapeutic potential of MSCs in this field. The application of modified MSCs in the treatment of stroke has demonstrated safety and feasibility in moderate to severe stroke patients [15, 16]. The safety profile and feasibility of MSCs as a therapeutic option are bolstered by well‐established harvesting methods, low risk of tumorigenicity, and high immune tolerance [17, 18]. These properties make MSCs a promising candidate for applications in stem cell therapy for stroke treatment. Initial successes of clinical trials drive the momentum to further explore and refine the therapeutic use of MSCs and potentially expand their use in routine clinical practice.

### 2.2. NSCs

NSCs are pivotal in neurobiology due to their self‐renewing and self‐replicating capabilities, enabling them to differentiate into neurons, astrocytes, and oligodendrocytes [19, 20]. These cells have dynamic roles across different stages of human development and throughout adulthood. In developmental stages, NSCs line the neural tube and form the central nervous system [21]. They engage in symmetric and asymmetric divisions, which generate intermediate progenitor cells that mature into the main cell types of the nervous system. The role of human‐specific NSCs in the outer subventricular zone (SVZ) is critical for cortical expansion during early development. In the adult brain, NSCs remain quiescent but can activate under specific conditions. Evidence suggests that this activation is highly regulated, with neurogenesis primarily confined to the hippocampus, a brain area crucial for learning and memory [21, 22]. Studies in rodent models have shown that NSC‐driven neurogenesis is modulated by intrinsic genetic programs and extrinsic environmental factors, varying significantly with the organism’s age and health conditions [20, 21]. Physiological stressors such as epilepsy, ischemia, and physical injuries can induce NSCs to enter active neurogenesis, potentially contributing to brain repair mechanisms. However, the natural capacity of NSCs to generate sufficient new cells is limited, underscoring NSC transplantation’s therapeutic potential [21, 22]. This approach aims to augment the brain’s intrinsic regenerative capacity, particularly following ischemic events where the demand for new neural cells exceeds the supply generated by endogenous NSCs. Despite the promise, translating the neurogenic potential of NSCs into effective clinical treatments requires overcoming numerous challenges, including optimizing cell survival, integration, and functional recovery posttransplantation, making NSC transplantation a focal point of ongoing research in regenerative medicine and neurotherapy [20, 22].

The utilization of NSC transplants in treating IS is compelling, largely due to their innate ability to migrate toward neurogenic sites and facilitate neurogenesis and vascularization [23]. This potential was illustrated in a Phase I clinical trial conducted by Kondziolka’s team in 2000, which demonstrated successful integration and terminal differentiation of tumor‐derived NSCs into neurons, with subsequent preclinical studies confirming differentiation into astrocytes [23]. This trial showed significant improvements in functional outcomes, although the results were not as pronounced in a subsequent Phase II trial led by the same team. Concerns arose regarding using malignant human tumor cells as sources for these cell lines, prompting a shift toward safer alternatives, including fetal porcine cells and human primary fetal brain‐derived NSCs. Current NSC transplant strategies explore allogeneic and xenogeneic sources; however, xenografts carry an elevated risk of immune rejection [23]. Moving forward, research is focused on developing sustainable and safe sources of NSCs, such as those derived from iPSCs, with an ongoing emphasis on enhancing the safety and efficacy of NSC transplants in clinical settings [23, 24]. This evolution reflects a commitment to refining regenerative therapies that can effectively mitigate the complex aftermath of IS.

### 2.3. iPSCs

**Figure 1:**
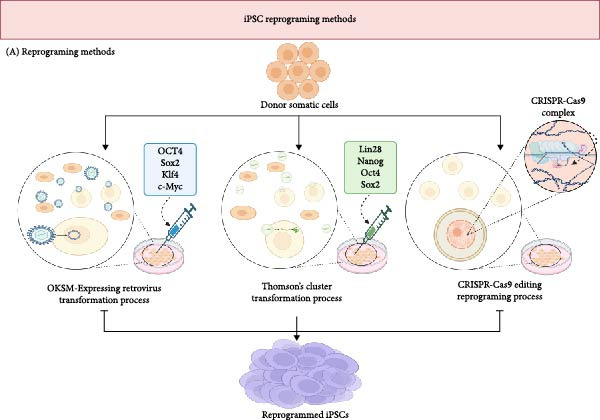
Figure represents the current mechanisms of somatic cell reprogramming into iPSCs. The cells can be reprogrammed via usage of combinations of OKSM and Thompson’s cluster transcription factors, or gene editing via CRISPR‐Cas9.

Somatic cells can be reprogrammed into iPSCs using several established approaches, summarized in Figure 1. The process was introduced by Takahashi and Yamanaka in 2006, representing a pivotal advancement in regenerative medicine by reprogramming mouse and human fibroblasts into cells with embryonic stem cell‐like properties [25, 26]. They achieved this by introducing four key transcription factors—Oct3/4, Sox2, Klf4, and cMYC, collectively referred to as the OSKM transcription factors, using retroviral vectors (Figure 1). Later, Yu’s team in 2009 enhanced the reprogramming efficiency by introducing Thomson’s cluster, which includes Lin28, Nanog, Oct4, and Sox2, diversifying the toolkit for generating iPSCs [25, 26]. The field has since evolved to multiple reprogramming vectors being explored, each presenting distinct advantages and drawbacks. Among these, nonintegrative vectors such as Sendai viruses, minicircles, and adenoviruses are preferred for clinical applications due to their reduced risk of disrupting host DNA, which mitigates the risk of tumorigenesis—a critical concern with OSKM factors where integration into the host genome can occur [27]. Moreover, the cutting‐edge CRISPR/Cas9 technology facilitates the reprogramming of somatic cells into iPSCs (Figure 1). It has also been utilized to correct genetically driven anomalies in synaptically active neurons from patients with Huntington’s disease [25]. Despite these advances, the application of CRISPR/Cas9 in iPSC technology is still under rigorous investigation to fully understand and reduce associated risks such as off‐target mutagenesis and residual tumorigenic potential [25]. This research of iPSC technology and its profound implications for future therapeutic strategies is ongoing, particularly those targeting complex neurological disorders.

While the potential of iPSCs in treating IS remains underexplored, their efficacy has been demonstrated in other domains, such as in the treatment of Parkinson’s disease in nonhuman primates and potential applications for Alzheimer’s disease [27]. In cardiac research, primate studies have shown that allogeneic iPSC transplants enhance cardiac contractile function following myocardial infarction, though they may predispose the heart to arrhythmias [25]. A challenge in the clinical application of iPSCs lies in the risk of teratoma formation, a concern attributable to the oncogenic potential of transcription factors like Klf4 and cMyc used in reprogramming [25, 26, 28]. Additionally, immune rejection presents a formidable obstacle, as iPSCs can provoke T cell‐dependent immune responses due to epigenetic discrepancies between the source and derived cells [25, 27]. However, these challenges are potentially surmountable with ongoing advances in gene‐editing technologies such as CRISPR/Cas9, which not only enhance the efficiency of reprogramming methods but might also allow for the modification of HLA phenotypes to reduce immunogenicity [25, 27]. The versatility of iPSCs is one of their most compelling attributes; they can be differentiated into various cell types, including MSC and NSC. They are derived from patient‐specific somatic cells, offering a personalized approach to regenerative medicine [25, 27, 28]. Despite these advantages, manufacturing techniques’ safety, consistency, and scalability remain issues that need to be addressed to advance iPSCs from the laboratory to clinical settings. As research progresses, these areas are key focal points to ensure that iPSC‐based therapies can be safely and effectively translated into clinical trials and future therapeutic applications.

## 3. Mechanisms of Stem Cell Therapy in IS

Stem cell‐based therapies exert their therapeutic effects in IS through multiple mechanisms that include immunomodulation, angiogenesis, neuroprotection, and neurogenesis. These actions are largely paracrine‐mediated and shared though MSCs NSCs, and iPSCs, stemming from the secretion of trophic factors such as VEGF, BDNF, TGF‐*β*, and other cytokines and chemokines [10–13, 22, 26, 29]. Collectively, these signals stabilize the BBB, attenuate neuroinflammation, promote angiogenesis, and enhance neuronal survival and differentiation. The effect of these processes contributes to reduced infarct volume, enhanced neuroplasticity, and improved functional recovery following ischemic injury. However, each stem cell type offers a unique combination of factors that contribute to their therapeutic functions outlined in the next sections.

### 3.1. MSC Therapeutic Mechanism

The secretome of MSCs is central to their regenerative capacity, driving immune modulation, angiogenesis, and neuroplasticity [10–13]. Following ischemic injury, MSC transmigration is guided by chemotactic signaling between SDF‐1 and expression of surface receptor CXCR‐4 [11–13]. Notably, MSCs that co‐express CXCR‐7 show further enhanced chemotactic responsiveness to SDF‐1 [11–13]. MSCs concurrently secrete factors that preserve BBB integrity, including downregulation of aquaporin‐4, inhibition of matrix metalloproteinase‐9 (MMP‐9), and TGF‐*β*‐mediated suppression of monocyte chemoattractant protein‐1 (MCP‐1), thereby minimizing endothelial damage and leukocyte infiltration [11–13].

MSCs immunomodulatory activity is exhibited through the secretion of TGF‐*β* and IL‐10. These factors work to decrease macrophage infiltration, inactivate microglia and astrocytes, and reduce tumor necrosis factor‐*α* (TNF‐*α*) levels [10, 12, 13]. These stem cells also modulate the IL‐17 and IL‐23 proinflammatory pathways, effectively inhibiting Th17 cell differentiation and survival. MSCs uniquely secrete CX3CL1, which facilitate the transition of microglia from a proinflammatory (M1) to a neuroprotective (M2) [10, 12, 13].

**Figure 2:**
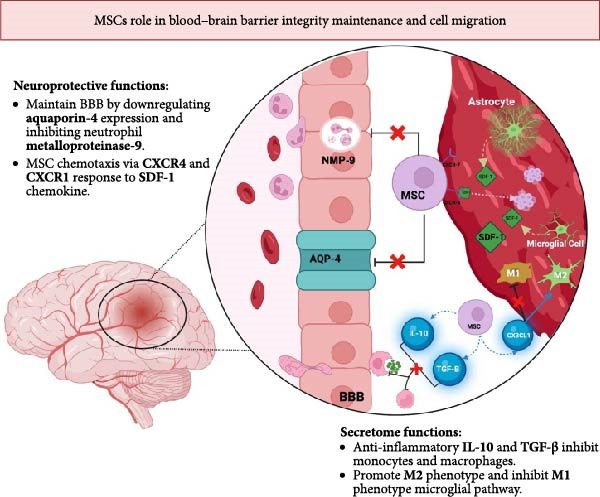
Figure depicting the BBB protective and migratory abilities of MSCs in an infarcted region of the brain. MSCs maintain BBB integrity by downregulating aquaporin‐4 and neutrophil metalloproteinase‐9. Astrocytes and microglial cells secrete SDF‐1 enabling chemotaxis of MSCs toward the infarct zone. The MSCs secretome promotes anti‐inflammatory microglial cell (M2) differentiation and downregulates monocyte and macrophage differentiation.

Angiogenesis and neuroprotection are directed by the release of VEGF, Ang‐1, and bFGF. This combination of factors enhances microvessel density and improves perfusion in ischemic regions [10, 12, 13]. The expression of VEGF and Ang‐1 is unique to MSCs and works synergistically as VEGF can transiently increase BBB permeability but is balanced by Ang‐1, which maintains vascular stability. Neurogenesis and neuroprotective functionality is largely driven by NGF and BDNF secretion, promoting neuronal survival and inhibiting apoptosis of neural progenitors. Furthermore, TGF‐*β*–dependent activation of endogenous NSCs promotes neuroblast migration and integration, underscoring the predominantly paracrine nature of MSC‐mediated recovery [10, 12, 13]. The multifaceted therapeutic mechanisms of MSCs are summarized in Figure 2.

### 3.2. NSC Therapeutic Mechanism

NSCs contribute to brain tissue repair through similar mechanisms as MSCs but are unique due to their inherent neural lineage differentiation potential. NSCs immunomodulatory function is mediated via the secretion of BDNF, NGF, and glial‐derived neurotrophic factor (GDNF), which suppress proinflammatory cytokines such as TNF‐*α*, IL‐1*β*, IL‐6, and MCP‐1, thereby preserving BBB integrity and reducing secondary injury, limiting infarct expansion [22, 29].

**Figure 3:**
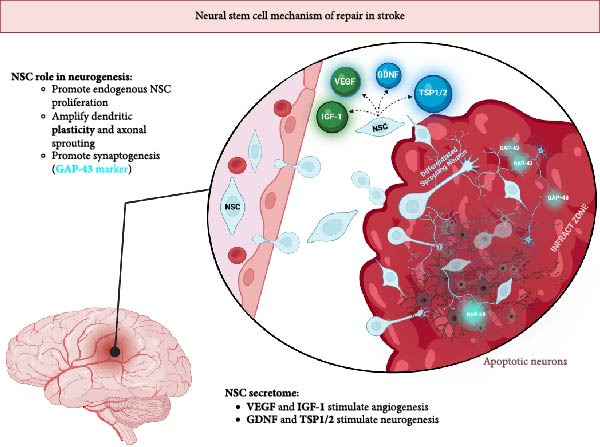
Figure depicting the neuromodulatory functions of transplanted neural stem cells (NSCs). Expression of GAP‐43 indicates active synaptogenesis. NCSs actively divide and replace apoptotic neurons while promoting dendritic plasticity and axonal sprouting. NSCs promote neurogenesis (blue) and angiogenesis (green) via secretion of GDNF, TSP1/2, IGF‐1, and VEGF.

Neurogenesis and angiogenesis are promoted through the coordinated release of VEGF, GDNF, IGF‐1, and thrombospondins‐1/2 (TSP1/2), increasing microvessel density and facilitating neuroblast migration toward areas of ischemic damage [22, 29]. The expression of TSP 1 and 2 is unique to the therapeutic functions of NCS in neurogenesis. Combined, the paracrine actions enhance dendritic plasticity, axonal sprouting, and synaptogenesis, evidenced by increased expression of GAP‐43, a marker of synaptic remodeling. NSCs can readily differentiate into neurons and glia, a process that is distinctive to these stem cells, permitting them to directly replace the apoptotic neurons. However, this process occurs over extended periods and is considered secondary to the trophic and modulatory effects of their secretome [22]. Figure 3 summarizes the therapeutic mechanisms unique to NSCs.

From a translational standpoint, NSCs pose relatively few ethical concerns compared to pluripotent sources, yet their scalability and donor‐dependent variability remain limiting factors for broad clinical deployment.

### 3.3. iPSCs Therapeutic Mechanism

iPSCs are the most versatile and ethically favorable platform for stroke therapy, combining the regenerative capabilities of MSCs and NSCs with a slightly expanded differentiation repertoire. iPSCs can give rise to NSC‐like populations, including neurons, astrocytes, and oligodendrocytes, which have demonstrated efficacy in restoring white matter integrity, enhancing cerebral perfusion, and improving metabolic function in preclinical stroke models [26]. The immunomodulatory effects of iPSCs mirror those of MSCs, characterized by increased levels of IL‐4 and IL‐10 and decreased expression of IL‐1*β*, TNF‐*α*, IL‐6, and IL‐2 [26, 28]. This favorable cytokine shift stabilizes the BBB and attenuates microglial activation after ischemic injury.

iPSC‐mediated angiogenesis and neuroprotection are supported by VEGF secretion and activation of STAT3‐dependent autophagy pathways, which enhance oxygenation and promote neuronal survival [26]. Human iPSC‐derived neuronal grafts have exhibited functional integration within host circuitry, forming synaptic connections and participating in signal transmission while reducing glial scar formation [26, 30, 31]. Like NSCs, iPSCs are capable of differentiating into mature neurons, however to a lesser extent [26]. Ongoing clinical trials continue to assess the safety, immunogenicity, and long‐term efficacy of iPSC‐based interventions. Notably, these cells show promise as a scalable and ethically sound strategy for stroke recovery [26, 30, 31].

## 4. Administration of Stem Cells

**Figure 4:**
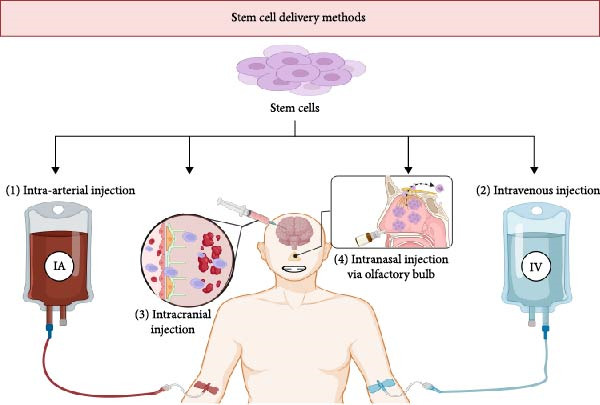
Systemic (IV, IA) and local (IC, IN) stem cell delivery methods.

The route and timing of stem cell delivery are critical determinants of therapeutic efficacy in stroke, with administration occurring either systemically or locally. Figure 4 provides an overview of current administration routes and their respective advantages and limitations. Timing is especially important: the greatest reductions in infarct volume and improvements in function are observed when stem cell transplantation occurs within the first 24 h and no later than 72 h poststroke [32]. Transplants administered within 0–8 h yield the most marked reduction in infarct size, while those delivered at 24 h still show behavioral improvements [32]. By contrast, treatments in the subacute and chronic phases (7–30 days poststroke) generally produce weaker outcomes [32]. These findings underscore the time‐sensitive nature of stem cell therapy and the need to refine therapeutic windows to optimize clinical benefit.

Systemic delivery methods include intravenous (IV) and intra‐arterial (IA) routes. IV administration is most common due to its simplicity but yields poor brain biodistribution, as roughly only 1% of cells reach cerebral tissue, with most sequestered in peripheral organs [23, 33]. This dispersion delays therapeutic action and increases off‐target effects, a significant limitation in the context of acute stroke. IA delivery provides more targeted access by infusing cells into the internal carotid or middle cerebral artery, allowing faster arrival to brain tissue with smaller doses [23, 33]. Animal studies show superior neurological outcomes with IA compared to IV or intracerebral (IC) routes, yet IA still yields only 0.6% of cells to the brain and carries a thrombosis risk [23]. Despite low engraftment rates, therapeutic effects are largely attributed to paracrine signaling—growth factors, cytokines, and chemokines secreted by transplanted cells—rather than direct cell replacement [33]. These findings illustrate the trade‐offs of systemic delivery and the need to enhance precision while maintaining safety.

Local methods include IC, intrathecal (IT), and intranasal (IN) administration. IC delivery results in the highest cell retention with approximately 60% at the lesion site, minimizing off‐target distribution and maximizing local therapeutic impact [23, 33]. However, IC injections are highly invasive and require specialized neurosurgical expertise, increasing procedural risks. IT delivery provides access to the cerebrospinal fluid and may enable broader distribution throughout the CNS, although the mechanisms of cell migration from CSF into brain tissue remain poorly defined. IN administration offers a non‐invasive alternative by utilizing the olfactory and trigeminal neural pathways to bypass the BBB [34]. While promising, the IN route requires further validation in clinical trials to determine efficacy and safety [23]. Ultimately, the choice of delivery route depends on clinical objectives, patient status, and temporal treatment window.

Adverse effect distribution varies by delivery route. IV administration showed the highest percentage of adverse effects (65.42%), followed by IC (28.53%) and IA (6.03%) [33]. However, these totals do not account for group sizes and may not fully represent relative risk. Importantly, IC administration was associated with the largest proportion of serious adverse effects (44.44%), exceeding both IV (17.24%) and IA (6%) [33]. This likely reflects the increased risks of IC injections, including bleeding, infection, and procedural injury to brain tissue. These findings highlight the need for balancing efficacy with procedural safety when selecting delivery methods.

Advancements in biomaterials aim to further improve stem cell delivery methods. Cell modification with hydrogels, nanoparticles, and scaffoldings has shown to increase cell survival and integration. Hydrogels provide a three‐dimensional scaffold that protects transplanted cells and enables sustained release of trophic factors within the infarct zone [35, 36]. By mitigating hypoxic stress, hydrogels support angiogenesis and neurogenesis [36]. Nanoparticles serve as delivery adjuncts, enhancing cell targeting and enabling controlled release of growth factors or gene modulators to promote tissue repair [37]. Other biomaterial constructs—such as scaffolds, microspheres, and encapsulation systems—can improve engraftment and survival by counteracting the hostile poststroke microenvironment [36, 38]. Fibrin matrices and collagen sponges have been used as injectable plugs to secure cells within infarct cavities and enhance adherence and differentiation [38]. Future work should focus on composite delivery systems, such as nanoparticle‐loaded hydrogels, to explore synergistic effects and improve therapeutic outcomes. As stem cell administration strategies evolve, continued research into biomaterial‐assisted delivery may significantly enhance the precision, safety, and efficacy of stem cell‐based stroke recovery.

## 5. Stem Cell Clinical Trial History

While preclinical evidence has demonstrated encouraging regenerative, neuroprotective, and immunomodulatory effects of stem cell therapies, their clinical translation has required careful evaluation through human trials. Multiple stem cell types have now been tested in IS patients using different delivery routes, dosages, and poststroke time windows. These trials have primarily focused on establishing safety and feasibility, with early indications of therapeutic benefit. Table 1 provides an overview of recent clinical trials, summarizing key study designs and outcomes to contextualize the current state of translation. The following sections outline the clinical progress of hematopoietic, mesenchymal, and neural lineage stem cells, detailing major findings and the translational barriers that remain.

**Table 1:** Clinical trials using stem‐cells for treatment of stroke.

NTC number	Status	Phase	Methods	Outcomes
02605707	Completed	I/II	Recruitment: 18 patients aged 18–80 with onset of IS symptoms up to 7 days prior. Of those, 6 received autologous MSCs, 6 autologous EPCs, and 6a placebo [39]. Cell administration: Patients received two 2.5 mln cells/kg IV injections a week apart from either autologous MSCs, EPCs, or placebo [39].	The study demonstrated the safety of IV autologous EPC and MSC transplants. There were no significant differences noted between the three groups other than an improvement in the Scandinavian Stroke Scale noted in the EPC group [39].

00473057	Completed	I	Recruitment: 32 patients aged 18–75 with onset of IS symptoms up to 90 days prior [40]. Cell administration: 10 patients received an IA injection of 500 mln MSCs, while 5 patients received an IV injection of 500 mln MSCs [40].	The study demonstrated the safety of both IV and IA autologous stem cell transplantation.

00875654	Completed	II	Recruitment: 31 patients aged 18–70 with onset of IS symptoms up to 6 weeks prior [16]. Of those, 20 received autologous MSCs and 11 placebo. Cell administration: In the treatment group, 10 received a low‐dose 100 mln MSCs IV transplant, and 10 received a high‐dose 300 mln MSCs IV transplant [16].	The study demonstrated the safety and feasibility of MSCs transplants for treating moderate and severe stroke [16]. The study noted improvements in behavioral and physiological motor outcomes especially during the initial 6 months of treatment [16].

01468064	Completed	I/II	Recruitment: 52 patients aged 30–75 with onset of IS symptoms up to 7 days prior. Of those, 16 received the autologous MSC transplant and 36 the placebo [41]. Cell administration: The treatment group received an IV autologous transplant of 50 mln MSCs 4 weeks following bone marrow aspiration [41].	The study demonstrated the safety and feasibility of IV MSC transplants [42]. The treatment group showed functional improvement and reduced mortality over the control group at the 5 year follow‐up [41].

02813512	Completed	I	Recruitment: 3 patients aged 65–80 suffering from chronic stroke, 6 months to 10 years post‐onset of symptoms. All 3 patients received brain transplants of GXNPC1 adipose‐derived stem cells (ADSCs) [42]. Cell administration: The treatment group received an IC autologous transplant of 100 mln ADSCs [42].	The study demonstrated the safety and feasibility of the IC ADSCs transplant. The treatment group reported no adverse effects and significant improvements at the 6 month follow‐up [42].

02378974	Completed	I/II	Recruitment: 18 patients aged 18–80 with onset of IS symptoms up to 7 days prior. Of those, 9 were assigned to Cohort 1 and 9 to Cohort 2 [23]. Cell administration: Cohort 1 received one IV Cordstem‐ST 200 mln transplant or placebo on day 0. Cohort 2 received two IV Cordstem‐ST 200 mln transplant or placebo on days 0 and 7 [23].	The study has been completed, and no adverse effects have been noted. Statistical analysis of the results is not currently available [23].

02961504	Completed	II/III	Recruitment: 206 patients aged 20–84 with onset of IS symptoms between 18–36 h prior. Of those 104 received the MultiStem transplant and 102 the placebo [43]. Cell administration: The treatment group received an IV injection of 120 mln MSCs [43].	The study demonstrated the safety of the MultiStem transplant; however, no significant improvements were noted between the treatment and placebo groups [43].

04631406	Recruiting	I/II	Recruitment: Recruiting patients aged 18–75, 6–60 months postischemic subcortical stroke [44]. Cell administration: Treatment group patients receive an IC injection of either 2.5, 5×, 10, or 2 mln NSCs [44].	Early results of the 6 transplanted patients suggest that the IC transplantation of NSCs is safe with noted motor function improvement 1–6 months posttreatment. Study is ongoing [44].

03570450	Recruiting	Ia/Ib	Recruitment: 95 patients total are recruited. Phase Ia recruited 15 patients over the age 18 to assess toxicity of IV ADSCs transplants. Phase Ib recruited 80 patients over the age 18 to assess dose effects of IV ADSCs transplants [45]. Cell administration: Treatment groups will receive an 4 IV injection of either 1.1, 2.1, 2.5, or 3.1 mln ADSCs, or a placebo [45].	Study is ongoing with expected primary outcome completion in 2025.

05850208	Recruiting	I	Recruitment: The study is recruiting 60 patients aged 18–65 in the rehabilitation period of cerebral infarction. Cell administration: The treatment group will receive 2 single IV transplants of 1 mln cells/kg body weight 1 week apart. The control group will continue standard treatment only.	Study is ongoing with no published data.

05008588	Recruiting	I/II	Recruitment: The study aims to recruit 15 participants aged 25–60 diagnosed with ischemic stroke in the acute phase. Cell administration: Patients in CM and UC‐MSCs experimental group receive both 3 daily doses of 3 cc of conditioned medium and one intraparenchymal injection of 20 mln umbilical cord MSCs. Patients in the UC‐MSC only group receive only the 20 mln UC‐MSC intraparenchymal injection. Patients in the active control continue with standard drug treatment.	Study is ongoing with no published data.

05292625	Recruiting	I/II	Recruitment: The study aims to recruit 48 participants aged 40–75 with neurological complications post‐IS. Of those 48, 16 participants will receive an IV injection of UC‐MSCs, 16 will receive intrathecal infusions, and 16 in the control group. Cell administration: Both experimental groups will receive two dosages of 1.5 mln cells/kg body weight in a 3 month intervention interval. The control group will continue with regular stroke treatment.	Study is ongoing with no published data.

04093336	Recruiting	I/II	Recruitment: The study aims to recruit 120 participants ages 18–80. Of those, 20 will be assigned to the Phase I trial to assess safety of the protocol. Phase II will follow the successful conclusion of Phase I and involve 100 with onset of infarction up to 24 h prior. Cell administration: In both phases, the treatment group receives a single dose IV injection of 2 mln MSCs/kg, the control group receives a single dose IV injection of placebo.	Study results are not published.

06129175	Recruiting	II/III	Recruitment: The study aims to recruit 80 participants over 18 years old with onset of acute IS within 4 weeks. Of those, 40 will be placed in the Neuroncell‐EX treatment group, and 40 in the control group. Cell administration: The intervention group will receive two single doses of an IV infusion of 2 mln UC‐MSCs/kg body weight on days 1 and 14. The control group will receive two single IV injections of saline on days 1 and 14.	Study is ongoing with no published data.

### 5.1. Hematopoietic Stem Cells (HSCs)

HSCs have been evaluated in Phase I, Phase I/II, and Phase II clinical trials for IS. In a 2009 Phase I study, Suarez‐Monteagudo et al. [46] administered 14–55 million autologous bone marrow mononuclear cells (BMSCs) IC to patients 1–10 years poststroke, reporting safety, tolerance, and modest neurological improvements, though the small sample size limited interpretation [23]. Subsequent trials by Savitz et al. [47] and Prasad et al. [48] used IV administration of 70–100 million cells/kg and 80 million BMSCs, respectively, delivered within 24–72 h or 7–30 days poststroke [23]. Both confirmed safety and feasibility. IA BMSC administration was investigated by Fredrich et al. [49] and Rosado‐de‐Castro et al. [50], but biodistribution to the brain was low [23]. A larger Phase II study by Prasad et al. [51] injected 280 million BMSCs intravenously in 120 patients within 7–30 days poststroke confirmed safety but showed no therapeutic benefit in moderately severe subacute stroke [23]. A Phase I/IIa trial by Taguchi et al. [52] compared 250 million vs. 340 million IV BMSCs, finding both doses safe, with the higher dose improving cerebral blood flow and metabolism at 6 months [23, 51]. The TREASURE Phase II/III randomized trial further evaluated IV MultiStem but showed no significant benefit at 90 days, highlighting persistent translational challenges [43]. Ongoing trials aim to refine dosing strategies and patient selection to determine whether HSCs can provide clinical benefit beyond safety.

### 5.2. MSCs

MSCs have undergone extensive clinical evaluation in IS. The first Phase I/II trial [53] (NCT00174323) delivered two IV injections of 50 million autologous MSCs shortly after stroke, demonstrating safety and functional improvement at 3, 6, and 12 months [23, 41, 53, 54]. A 5‐year follow‐up study from the same group reported sustained benefit and reduced mortality. A Phase I/II protocol by Bhasin et al. [54] (NCT01297413) using 50 million serum‐free MSCs confirmed safety but showed no significant functional improvement over controls. Fang et al. [55] (NCT01438593) compared autologous MSCs against endothelial progenitor cells using a two‐dose injection of 2.5 million cells/kg but found no clinical benefit, likely due to the small sample size [23]. To utilize MSCs’ low immunogenicity, the MASTERS Phase II trial (NCT01436487) evaluated allogeneic MultiStem (400 million or 1.2 billion cells) administered within 24–48 h poststroke. While safety was established, efficacy was not demonstrated, leading to the ongoing MASTERS‐2 Phase III trial (NCT03545607) that administers a single 1.2‐billion‐cell dose and assesses outcomes over 365 days [23, 39]. The TREASURE trial (NCT02961504) followed a similar protocol but showed no significant benefit at 90 or 365 days, potentially influenced by an older patient cohort [43]. Collectively, these studies show that MSC transplantation is consistently safe but requires further optimization of dosing, manufacturing methods, and patient stratification to demonstrate efficacy in IS.

### 5.3. Neural Lineage Stem Cells

NSC transplantation for chronic stroke was first explored by Kondziolka et al. [56], using 2–6 million NT2/D1 teratocarcinoma‐derived cells injected IC into patients 6 months–6 years poststroke [23]. The procedure was safe and associated with neurological improvements at 6 months. A follow‐up Phase II study increased the dose to 5–10 million cells across 14 patients but yielded limited outcomes, with benefit observed primarily in upper limb motor testing [23, 57]. Oncogenic risk associated with tumor‐derived NT2/D1 cells prompted exploration of xenogeneic fetal porcine NSCs, but a Phase I trial was halted due to seizure adverse effects and symptom worsening in two patients [23, 58]. The PISCES trials marked major progress. Using the CTX0E03 human fetal cortical cell line, PISCES 1 (Phase I) evaluated IC administration of 2.5–20 million NSCs in male patients 6–24 months poststroke [23, 59]. Concerns about estrogen‐related c‐myc activation initially restricted enrollment to males. PISCES 2 expanded eligibility to include females and administered 20 million cells to patients 2–13 months poststroke. Improved upper limb function was reported at 3, 6, and 12 months—but only in patients with initial arm movement [23, 60]. PISCES 3 aimed to expand to U.S. sites but was discontinued as the sponsor shifted strategic priorities [60]. The cumulative data support NSC safety and functional potential, though outcomes remain inconsistent. iPSC‐derived NSCs represent a promising next step, offering scalable, ethically sourced alternatives that may overcome current limitations in sourcing, immune compatibility, and tumorigenicity.

## 6. Recommendations for Future Research: STEPS

The field of stem cell research is constantly growing and changing given the ongoing ethical and technological changes. The STEPS meetings serve as a critical platform where academic and industry leaders converge to exchange insights on the latest advancements in cell therapy for stroke and provide recommendations for future preclinical and clinical research. The primary focus of the fourth STEPS meeting was to explore strategies for accelerating the development of cell therapies for stroke while meticulously safeguarding patient safety [61].

### 6.1. Updated Guidelines for Future Preclinical Trials

In these discussions, the assembled experts highlighted the importance of utilizing large animal models (LAMs) in preclinical trials to better understand the effects of stem cells on the brain. LAMs are particularly valued for their anatomical and physiological similarities to humans. They include a comparable gray‐to‐white matter ratio and sufficient size that facilitates more realistic cell migration and distribution studies. This makes them exceptionally suitable for clinical imaging applications and for modeling the complexities of human neurological structures [61]. Furthermore, LAMs are considered essential for validating the safety and efficacy of proposed treatments, offering more relevant data on potential cognitive impairments resulting from stroke. The guidelines also emphasize the necessity of diversifying trial subjects to include various sexes, ages, and comorbidities, thus reflecting the broad spectrum of the human population affected by stroke [61]. This approach ensures that the outcomes are robust and generalizable across different demographic groups. Another critical recommendation is the examination of drug–cell interactions, acknowledging that stroke patients receive multiple medications concurrently. Future preclinical trials should, therefore, incorporate simultaneous administration of cell therapy and common poststroke medications such as antiplatelets, antihypertensives, and statins. This strategy is intended to enhance the relevance and applicability of the research findings to real‐world clinical settings [61]. Lastly, the integration of neurorehabilitation into preclinical protocols was advised. Since rehabilitation is a cornerstone of poststroke recovery, its inclusion in trial designs is vital for assessing the synergistic effects of stem cell therapy and rehabilitative practices, aiming to maximize recovery and improve the quality of life for stroke survivors [61]. These updated guidelines from the STEPS meeting are poised to significantly refine the trajectory of future research, ensuring that it is not only scientifically rigorous but also aligned with the practical realities of stroke treatment and patient care.

### 6.2. New Considerations for Preclinical Studies

The latest recommendations for preclinical studies in stroke treatment emphasize the need to broaden research paradigms to include a variety of stroke models, particularly those with smaller infarctions that closely mimic clinical scenarios. Experts suggest that stroke models targeting subcortical gray and white matter are particularly apt for treating stem cells like glial progenitor cells, which have inherent tissue repair capabilities [61]. Additionally, IC hemorrhage models are now considered promising targets for cell therapy. This shift is based on their potential to release therapeutic factors that can counteract the pathological mechanisms specific to hemorrhagic stroke. A crucial area of focus for these studies is the preconditioning of stem cells. By optimizing the conditions under which cells are cultured before transplantation, researchers aim to enhance the cells’ survival, long‐term engraftment, and functional integration into host tissues. This approach could improve the efficacy of stem cell therapies in clinical applications. Ensuring the safety of stem cell treatments remains paramount. Future preclinical studies are encouraged to rigorously assess both short‐term and long‐term safety profiles of stem cell therapies. This includes a thorough analysis of the biodistribution and persistence of transplanted cells in the host organism and their paracrine and immunomodulatory effects [61]. Furthermore, it is vital to meticulously evaluate potential adverse outcomes such as tumorigenesis, abnormal tissue growth, or aberrant ectopic fiber sprouting, particularly in cells characterized by high proliferation and differentiation potentials. These new considerations are designed to refine the development of stem cell therapies, ensuring they are effective in tissue repair and functional recovery poststroke and safe and reliable over the long term [61]. This comprehensive approach aims to facilitate more successful translations of preclinical findings into clinical trials and eventual therapeutic applications.

### 6.3. Ideas for Accelerating and Improving Preclinical Research

During the STEPS 4 meeting, experts discussed strategies for accelerating and enhancing preclinical research in cell therapy for stroke. A key recommendation was to delineate the objectives of early and later phases of clinical trials. Phase I/II trials should primarily focus on assessing the safety and feasibility of the therapies, establishing a foundation for more focused investigations. In contrast, later phases should pivot toward evaluating the efficacy of these interventions, ensuring that advancements in therapy development are both impactful and targeted [61]. To streamline this process, it was suggested that preclinical studies should simultaneously address safety concerns while tailoring efficacy tests. This approach would improve the timing of these studies and help identify the patient populations that are most likely to benefit from specific treatments. Such dual‐focused studies can expedite the transition from safety assessments to efficacy evaluations, enhancing the overall pace of research. Furthermore, the establishment of a comprehensive database was proposed. This database would catalog information on tissues that respond positively or negatively to treatments, providing invaluable data for refining patient selection criteria. Researchers can design more personalized and effective treatment protocols by understanding which patients are more likely to benefit from specific therapies [61]. Recognizing the financial burdens associated with extensive preclinical and clinical trials, the STEPS group strongly advocated for increased collaboration between academic institutions and the industry. By pooling resources and expertise, these partnerships can significantly reduce costs and leverage shared knowledge, driving the development of innovative cell therapies for stroke more efficiently. These strategic recommendations from the STEPS 4 meeting aim to accelerate the pace of preclinical research and enhance its quality, ensuring that new treatments are safe and effective [61]. Such advancements are crucial for pushing the boundaries of stroke therapy and improving patient outcomes in the long term.

## 7. Conclusion

Stem cell therapy for stroke occupies a promising yet challenging position at the forefront of regenerative medicine. Over the past two decades, research has demonstrated that stem cells can support neural repair and functional recovery through mechanisms including neuroprotection, immunomodulation, angiogenesis, and neurogenesis [14]. Their secretome remains central to these effects and represents a key target for enhancing therapeutic potency. Emerging strategies include integrating stem cell therapy with neurorehabilitation and engineering cells to overexpress neurotrophic factors for improved efficacy [62]. Advances in gene‐editing technologies such as CRISPR/Cas9 offer opportunities to improve safety and scalability, particularly for iPSCs. Refinements in cell modification and reprogramming protocols may enable development of universal donor cells and expand clinical accessibility.

**Figure 5:**
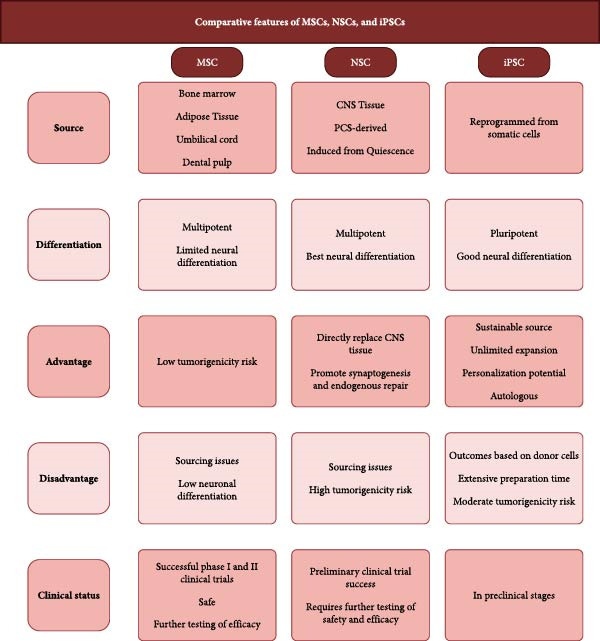
Figure summarizes key characteristics of MSCs, NSCs, and iPSCs relevant to their use in stroke treatment. Categories compared include cell source, differentiation capacity, therapeutic advantages, therapeutic disadvantages, and current clinical status.

While different stem cell types show therapeutic potential, their limitations critically shape clinical translation. MSCs face donor variability and heterogeneity, compromising reproducibility; NSCs raise ethical and sourcing concerns due to reliance on fetal or tumor‐derived lines; and iPSCs carry risks of tumorigenicity and high manufacturing costs. Figure 5 compares the core characteristics of MSCs, NSCs, and iPSCs, highlighting how their distinct strengths and limitations inform therapeutic selection. Importantly, evidence from mechanistic studies and clinical trials reveals that these cells exert their benefits primarily through paracrine signaling rather than direct neuronal replacement, which helps explain why biodistribution, inflammatory resistance, and cell survival within the ischemic microenvironment remain major barriers to efficacy. Clinical outcomes reflect these challenges, with low cell retention and variable responses limiting functional recovery, as evidenced in the TREASURE and MASTERS trials. Accordingly, the limitations of each cell type directly guide emerging strategies: standardized manufacturing protocols for MSCs, ethically sourced NSC alternatives, enhanced iPSC reprogramming and safety validation, genetic engineering to reduce immunogenicity and tumorigenicity, biomaterial cell enhancing modalities, and secretome‐based approaches to modulate the microenvironment. Moving forward, progress will rely not on discovering new cell types but on refining delivery, timing, dosing, and mechanistic precision, while integrating patient‐specific variables, drug–cell interactions, neurorehabilitation, and STEPS‐aligned frameworks to prevent premature trial failure and enhance clinical relevance.

As stroke incidence continues to rise in aging populations, advancing stem cell‐based therapies is an urgent clinical priority. This review integrates molecular mechanisms, clinical trial outcomes, and translational guidelines to bridge bench and bedside perspectives. This work aims to inform research trajectories that move the field toward more consistent methodologies, improved safety, and meaningful clinical impact.

## Ethics Statement

The authors have nothing to report.

## Consent

The authors have nothing to report.

## Conflicts of Interest

The authors declare no conflicts of interest.

## Funding

This research was supported by the Truman State University STEP grant and National Institutes of Health (Grants HL141998 and HL141998‐01S1) to S.M.; (Grants AA031465, AA025744, AA026708, and AA025744‐02S1) to M.P.; (Grants P20GM12130) to Christopher Kevil. Miriyala provided funding to pay the publication charges for this article.

## Data Availability

The authors have nothing to report.
